# Sleep after learning aids the consolidation of factual knowledge, but not relearning

**DOI:** 10.1093/sleep/zsaa210

**Published:** 2020-10-09

**Authors:** James N Cousins, Teck Boon Teo, Zhi Yi Tan, Kian F Wong, Michael W L Chee

**Affiliations:** 1 Centre for Cognitive Neuroscience, Duke-NUS Medical School, Singapore; 2 Donders Institute for Brain, Cognition & Behaviour, Radboud University Medical Centre, Nijmegen, The Netherlands; 3 Centre for Sleep and Cognition, Yong Loo Lin School of Medicine, National University of Singapore, Singapore

**Keywords:** memory, long-term memory, learning, relearning, declarative memory, consolidation, encoding

## Abstract

**Study Objectives:**

Sleep strengthens and reorganizes declarative memories, but the extent to which these processes benefit subsequent relearning of the same material remains unknown. It is also unclear whether sleep-memory effects translate to educationally realistic learning tasks and improve long-term learning outcomes.

**Methods:**

Young adults learned factual knowledge in two learning sessions that were 12 h apart and separated by either nocturnal sleep (*n* = 26) or daytime wakefulness (*n* = 26). Memory before and after the retention interval was compared to assess the effect of sleep on consolidation, while memory before and after the second learning session was compared to assess relearning. A final test 1 week later assessed whether there was any long-term advantage to sleeping between two study sessions.

**Results:**

Sleep significantly enhanced consolidation of factual knowledge (*p* = 0.01, *d* = 0.72), but groups did not differ in their capacity to relearn the materials (*p* = 0.72, *d* = 0.10). After 1 week, a numerical memory advantage remained for the sleep group but was no longer significant (*p* = 0.21, *d* = 0.35).

**Conclusions:**

Reduced forgetting after sleep is a robust finding that extends to our ecologically valid learning task, but we found no evidence that sleep enhances relearning. Our findings can exclude a large effect of sleep on long-term memory after 1 week, but hint at a smaller effect, leaving open the possibility of practical benefits from organizing study sessions around nocturnal sleep. These findings highlight the importance of revisiting key sleep-memory effects to assess their relevance to long-term learning outcomes with naturalistic learning materials.

**Statement of Significance**

Sleep is linked to a wide range of memory functions, but recent experiments have been unable to replicate some earlier claims. Additionally, few studies have assessed the outcomes associated with more realistic learning tasks and sleep schedules. Here, we show that sleep after learning educationally relevant factual knowledge provides an initial overnight boost. However, subsequent relearning of the same materials was not enhanced by prior sleep, and the initial memory consolidation advantage was reduced one week later. These findings do not support earlier claims that sleep alters memory representations to improve relearning. Further research is warranted to assess the practical relevance of reported sleep-memory effects.

## Introduction

There is a long-standing debate about how declarative knowledge is stored and evolves over time: from the initial processes of encoding, to the active mechanisms involved in offline strengthening and restructuring of memory representations (consolidation), and finally to the recapitulation of memories during retrieval. Consolidation has been the focus of a great deal of research, particularly with regard to processes during sleep that may optimize memory networks for long-term storage. It has been consistently shown that a period of sleep after learning results in better recall than when learning is followed by an equivalent period of wakefulness [1–6]. This sleep benefit is thought to occur through at least three different mechanisms. These comprise a systems consolidation process whereby memory reactivation reorganizes representations for long-term storage [5], a homeostatic process that prevents saturation of synaptic connections in memory networks [7], and the protection of memories from interference during sleep [8, 9]. Slow-wave activity (SWA) and sleep spindles during nonrapid eye movement (NREM) sleep have been highlighted as potential electrophysiological markers for these memory processes [5, 10–14].

Sleep has also been implicated in a wide range of qualitative alterations to declarative memories, lending support to the idea that sleep actively restructures the neural representations of memories [5]. These include findings that sleep facilitates the integration of new information into existing memory networks [12, 13], and abstracts patterns from recently encountered stimuli to facilitate generalization to new experiences or generate creative insights [15, 16]. However, the evidence for some of these claims has been recently questioned [17].

A handful of recent studies introduced a further learning and test session after sleep to examine whether sleep shapes new memories in a way that enhances subsequent relearning of the same material [1, 4, 18]. The first of these studies assessed memory for 20 Swahili-English paired associates encoded during an initial learning session and a subsequent relearning session 12 h later [1]. They found that when presented with the Swahili word, retrieval of the English translation was significantly enhanced when the 12 h interval included overnight sleep relative to when it comprised daytime wakefulness. However, sleep and wake conditions did not differ after an opportunity to relearn the paired associates, or during a test 10 days later. In sum, this study replicated the well-established benefit of sleep for memory consolidation [5], but found no evidence that sleep affects relearning. Mazza and colleagues used a similar design assessing memory for 16 Swahili-French paired associates after a 12 h interval including sleep or wakefulness [4]. They observed a sleep advantage for the initial test after the retention interval, while the sleep group also required significantly fewer trials to reach 100% correct recall during the relearning session. Moreover, the sleep group retained a sizeable memory advantage after 1 week, and 6 months. In contrast to the first study [1], these findings indicate that sleep strongly facilitates relearning. A third study found that while sleep had no immediate impact on the consolidation of category knowledge, it did improve participants’ ability to integrate visual and auditory domains to determine category structure during relearning [18]. Given these inconsistent findings, the boundary conditions where sleep impacts upon relearning remain to be determined.

Such relearning effects may result from strengthening [5] or protection [8] of memories during sleep, making representations easier to re-encode after waking. Alternatively, the abstraction of underlying patterns to form schematic representations during sleep [15, 16] could serve to enhance the encoding of the same or related information during relearning [12, 19, 20]. This subtle restructuring process is particularly relevant to the complex knowledge structures encountered in daily life, but is not easily probed with the paired-associate stimuli used in previous studies of sleep and relearning [1, 4].

Establishing the robustness and ecological validity of these sleep-related benefits on learning has important implications for education, for example, to determine whether study episodes should be arranged to occur close to sleep in order to optimize learning outcomes. There is currently a gap in our understanding of how laboratory-based sleep findings can be translated to daily life [21], both in terms of generalizing from laboratory tasks to the complex types of information typically learned in education, and in determining whether sleep effects extend beyond the initial days of learning [22]. A handful of studies have begun to explore these effects under more naturalistic conditions, with realistic learning tasks [23–25] and typical patterns of sleep [26, 27], but these effects remain poorly understood. For example, we recently examined the memory benefits of daytime naps using stimuli that simulated the practical and semantic context in which a large proportion of declarative learning takes place [25]. This “factual knowledge task” entailed learning about arthropods across several long episodes, including sentences alongside images, opportunities to recap specific facts, make notes, and engage in free recall to aid learning. Importantly, the arthropod species existed in a hierarchy of semantic relatedness, sharing some features and differing in others, while also relating to participants’ prior experience with similar species. This interdependence of knowledge—and the potential for memory interference and facilitation within it—is often removed from laboratory-based tasks, where the focus is on isolated items or pairs and the recall of specific episodes (e.g. do you remember this image from a prior session?). That is, encoded information shares an episodic context, but no meaningful semantic context. Examining memory effects under more naturalistic conditions is important not only to understand what happens to long-term memories in the context of education, but also in developing models of memory consolidation that may generalize to many different forms of declarative memory.

In light of this, the current study addressed three questions: (1) Does sleep benefit the consolidation of a naturalistic factual knowledge task? (2) Does sleep enhance the relearning of factual knowledge? (3) Does sleep between two study episodes enhance long-term memory 1 week later? We utilized the aforementioned factual knowledge task [24–26] suited for generalizing research findings to learning in education. These stimuli also encouraged the formation of schema [12], which may be an important factor in sleep-relearning effects.

**Figure 1.**
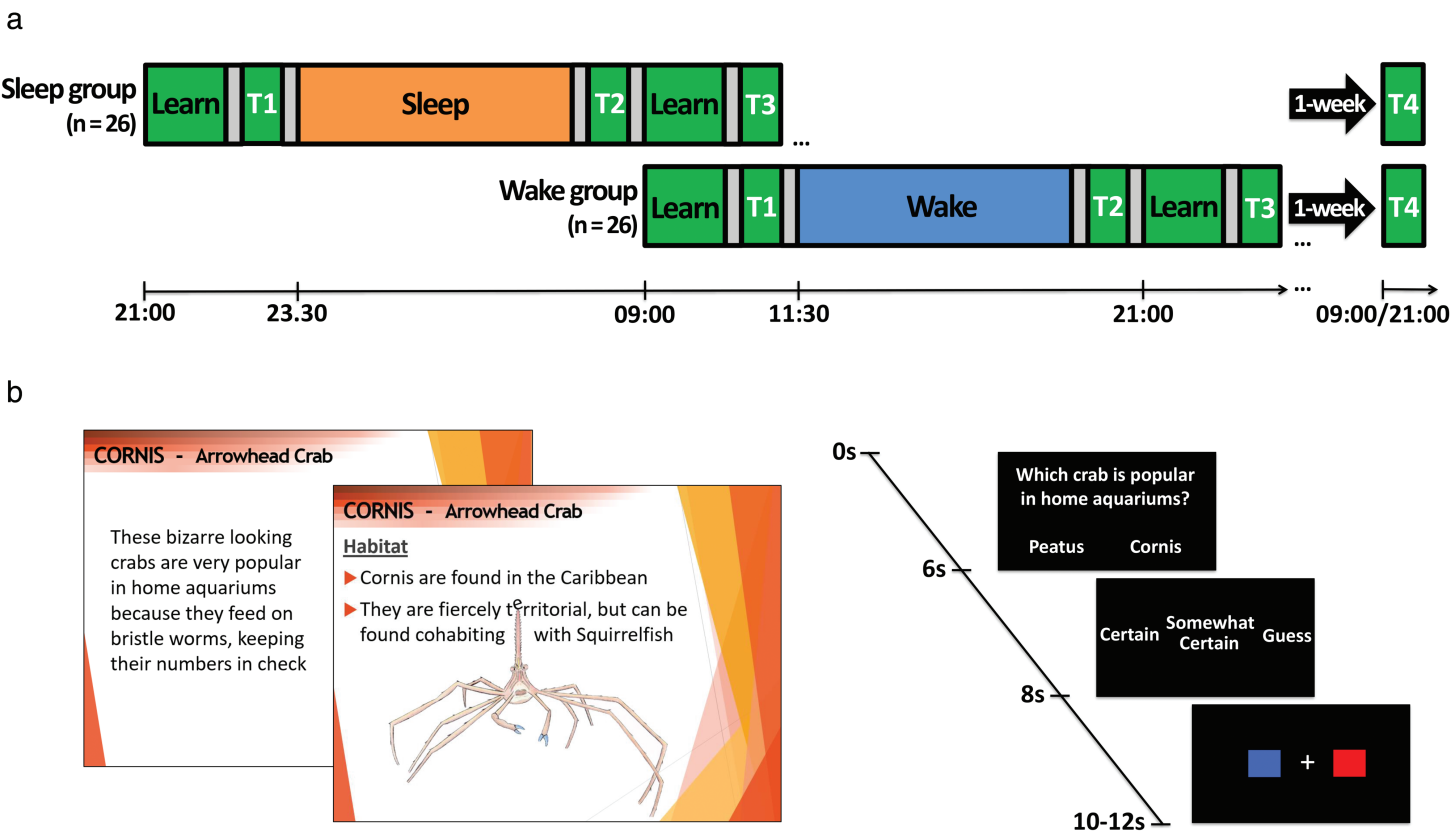
Protocol and stimuli. (A) Participants learned for 1 h 30 min with a 10 min break in between. The spacing of learning and tests was identical in the two groups, but the timing was shifted by 12 h. The first test (T1) indicated whether groups differed for initial encoding of factual knowledge. Comparison of T2 and T1 provided a measure of sleeps influence during the day or night retention interval. Relearning was assessed by comparing the test before the second learning session (T2) to the test afterwards (T3). The long-term benefit of these different learning schedules was assessed by comparing performance at the first test (T1) to a final test that occurred 1 week later (T4). (B) Self-paced learning consisted of detailed information about 12 arthropods presented on slides. Tests includes 90 two alternative forced choice questions of varying difficulty, followed by a confidence rating and baseline task where participants indicated the color of a central cross.

Participants learned detailed facts about arthropods across two sessions (1 h 30 min each; Table 1) that were separated by 12 h of either nocturnal sleep (sleep group) or daytime wakefulness (wake group; Figure 1). The first session included all of the materials about arthropods, and the second session assessed relearning by re-presenting the same materials again for an identical period of time. Knowledge was tested with two alternative forced-choice questions at four time points that followed the initial learning session (T1), the retention interval (T2), the relearning session (T3), and 1 week (T4). To address our three main questions, performance was contrasted to produce measures of consolidation (T2-T1), relearning (T3-T2), and long-term memory (T4-T1).

**Table 1.** Example stimuli for facts and questions

Fact	Question	Answer	Foil
Cornis live on coral reefs at depths of up to 20 m	What is the maximum depth for Cornis?	20 m	9 m
Oatii reside in shallow waters of 3–9 m because sunlight is crucial to the survival of corals that they carry on their backs	What is the maximum depth for Oatii?	9 m	20 m
The zoologist Johann Herbst first described Cornis (1788)	Hebst described which crab?	Cornis	Latro
Latro were first described by William Leach (1816)	Who first described Latro?	Leach	Hebst

Consistent with prior research [1–6], we predicted that consolidation would be significantly greater for the sleep group, while measures of slow-wave sleep and spindle activity in those who slept would positively correlate with consolidation. We also predicted that sleep would benefit relearning [4, 18], with the sleep group demonstrating greater improvement in performance after the second learning session. Finally, in line with a recent daytime nap study using the same factual knowledge task [25], we also predicted that the combined sleep-dependent enhancement to consolidation and relearning would remain after 1 week.

## Methods

### Participants

A total of 56 young adults were recruited via online advertisements from a sample of undergraduates who reported having no history of neurological, psychiatric, or sleep disorders and were taking no medication during the experiment. Participants were fluent English speakers and consumed <2 caffeinated beverages a day. Students taking biology and ecology majors were excluded. Participants provided written informed consent and received monetary compensation after completion, in accordance with a protocol approved by the National University of Singapore Institutional Review Board.

Four participants were excluded due to computer error during learning (*n* = 1) and overall memory at T1 that was close to chance and >2 *SD* below the mean (*n* = 3). The 52 participants that remained (19–28 years, age *M* = 22.56, *SD* = 2.68 years) were assigned to sleep (*n* = 26, 15 females, age *M* = 22.62, *SD* = 2.67 years) and wake (*n* = 26, 16 females, age *M* = 22.50, *SD* = 2.75 years) groups. Groups did not significantly differ in age, sex, Pittsburg Sleep Quality Index (PSQI), Morningness-Eveningness Questionnaire (MEQ) (Table 2), or several tests evaluating short-term memory, fluid intelligence, and verbal memory (*p* > 0.05; Supplementary Table S1).

**Table 2.** Demographics, sleep habits, and actigraphically assessed sleep

	Sleep		Wake			
	Mean	*SD*	Mean	*SD*	*t*	*P*
Age (y)	22.62	2.67	22.50	2.75	0.15	0.88
Pittsburgh Sleep Quality Index global score	4.19	2.32	3.96	1.79	0.40	0.69
MEQ score	49.85	12.00	50.17	9.88	0.10	0.92
Actigraphy						
TIB—mean of 3-days prior to experiment (h)	8.01	0.56	7.81	0.49	1.23	0.23
TST—mean of 3-days prior to experiment (h)	6.42	0.81	6.63	0.85	0.85	0.40
TIB—mean of retention interval (h)	7.90	0.66	8.06	0.48	0.85	0.40
TST—mean of retention interval (h)	6.55	0.75	6.78	0.76	0.98	0.36

y, year; *SD*, standard deviation; TIB, time in bed; h, hour; actigraphy threshold: medium.

### Design

Participants performed two learning sessions separated by 12 h, with four tests to assess memory at different delays (T1, T2, T3, and T4; Figure 1, A). The key manipulation was that the sleep group had polysomnography (PSG)-recorded nocturnal sleep between the two learning sessions, while the wake group had a day of wakefulness. This produced a mixed design with a between-subjects factor of group (sleep and wake) and a within-subjects factor of test (T1, T2, T3, and T4). Individual tests were compared to provide measures of consolidation (T2-T1), relearning (T3-T2), and long-term memory (T4-T1).

Psychomotor vigilance was assessed throughout the protocol to assess the influence of sleep on alertness, and a battery of cognitive tests were administered during a briefing to ensure groups were similar in their cognitive abilities. Actigraphy confirmed that participants were well-rested prior to experimental sessions.

### Materials

#### Cognitive tests

A battery of cognitive tests were administered during a briefing session 3–7 days prior to the experiment. These evaluated short-term memory (Digit Span; Weschler Adult Intelligence Scale, WAIS), fluid intelligence (12-item version of RAVENS Advanced Progressive Matrices), and verbal memory (Rey-Auditory Verbal Learning Test, RAVLT).

#### Factual knowledge learning

During the two learning sessions, participants encoded detailed facts about 12 species of arthropod via two 40 min blocks separated by a 10 min break. All of the information about all 12 species (6 ants and 6 crabs) was learned in the first session, and participants had an opportunity to relearn the exact same materials in the second session. During breaks participants were permitted to use smartphones, but were instructed not to peruse information about arthropods.

Learning blocks contained approximately 80 slides of factual information in the form of bullet points and images (Figure 1, B) presented with Matlab 2012 (Mathworks Inc., Natick, MA). Some slides instructed participants to write on paper what they could recall about species to assist long-term retention. Participants moved through slides at their own pace, but were instructed to observe a minimum speed to ensure all slides were seen, denoted by markers indicating how much time should have passed at 5 min intervals. A counter was visible throughout. The final slide of each block instructed participants to use remaining time to recap the information.

#### Factual knowledge tests

All tests involved two alternative forced-choice questions followed by a confidence rating (certain, somewhat certain, and guess; Figure 1, B). Questions were displayed until a response was made or when 6 s had elapsed. Confidence ratings were displayed until a response or when 2 s had elapsed. This was followed by a brief distractor task involving a white fixation cross flanked by a red box (left) and blue box (right). Following a pseudorandom delay, the cross became red or blue. Participants were instructed to press the button corresponding to the color. This task lasted 2–4 s, with target onset randomly generated to be 500 ms to 2 s from onset of the task, allowing at least 1 s to respond. The foil to each question was most often the answer to the same question for a different species. Participants were instructed to answer each question within the time frame and to make guesses if they did not know.

A pretest consisted of 60 questions of varying difficulty and was used to ensure that groups did not differ in prior knowledge of the to-be-learned materials. The main experimental tests (T1, T2, T3, and T4) comprised four sets of 90 questions matched for difficulty based on data from two prior experiments [24, 25]. These were pseudo-randomly assigned to tests and counterbalanced across participants. Each set was performed in separate blocks for questions about ants (45 questions) and crabs (45 questions), separated by a 30 s break with order counterbalanced.

#### Psychomotor vigilance task

A 10 min psychomotor vigilance task (PVT) was performed prior to each testing block. A counter appeared on screen at random intervals between 2 and 10 s, and participants responded with a key press as quickly as possible. Failure to respond within 10 s initiated an alerting tone. Lapses (responses >500 ms) were measured.

### Procedure

Participants attended a briefing 3–7 days prior to the main experimental session where they performed questionnaires and cognitive tests that were used to ensure groups were similar in terms of sleep history and cognitive capacity. Participants were provided with an actiwatch and sleep diary. They were instructed to keep to a sleep schedule for 3 nights prior to the experimental session and during the one-week retention interval (6.5–9 h sleep per night, sleep before 12:30 am, and wake before 09:00 am).

For the sleep group, participants arrived for the first session at the laboratory at 07:30 pm. They were fitted for PSG prior to performing the first PVT (08:50 pm–09:00 pm). Factual knowledge learning took place from 09:00 pm to 10:30 pm. This was followed by the second PVT (10:50 pm–11:00 pm) and T1 (11:00 pm–11:20 pm). Participants then slept in a laboratory bedroom (11:30 pm–07:30 am). Upon waking, PSG was removed and participants showered. The second session began with a PVT (08:10 am–08:20 am), followed by T2 (08:20 am–08:40 am). Participants then had breakfast during a 20 min break prior to factual knowledge relearning (09:00 am–10:30 am), a PVT (10:50 am–11:00 am), and T3 (11:00 am–11:20 am), after which participants could leave the laboratory.

This procedure was identical for the wake group, except that timings were shifted by 12 h (e.g. the first learning session began 09:00 am rather than 09:00 pm), there was no PSG and participants were free to leave the laboratory in the retention interval. Participants were instructed not to nap during this period, confirmed with actigraphy.

Both groups returned to the laboratory 1 week later for the final test (T4) in the MRI scanner (MR data not reported here), with half the participants from each group being tested in the morning and evening. Participants performed a final PVT immediately prior to entering the MRI scanner for T4 in the morning (10:10 am–10:40 am) or evening (10:10 pm–10:40 pm), after which they were reimbursed for their time and permitted to leave.

### Actigraphy

Participants wore an Actiwatch AW-2 (Phillips Respironics Inc., Pittsburgh, PA) for at least 3-days prior to the experiment and during the 1-week retention interval, alongside a sleep diary used to clean and verify the data. Data were scored with Actiware software (version 6.0.2) at 30-s resolution and medium sensitivity.

### Polysomnography

Sleep recordings were acquired with a 16-channel MR amplifier (BrainAmp, Brain Products, GmbH, Gilching, Germany) from 7 scalp derivations (F3, F4, C3, C4, Cz, O1, and O2) referenced to linked mastoids (A1 and A2), according to the 10–20 system. Electrooculogram (EOG), electromyogram (EMG), and forehead ground electrodes were also attached. Impedance was <5 kΩ for electroencepholography (EEG) and <10 kΩ for EOG and EMG. Signals were collected at a digital sampling rate of 500 Hz (bandpass filtered 0.1–250 Hz). Sleep was scored offline according to standardized criteria in 30 s epochs using the FASST-Z3Score toolbox [28] (https://github.com/amiyapatanaik/FASST-Z3Score) and checked by a trained technician. The following parameters were reported for participants in the sleep group: duration of NREM sleep stages (N1, N2, and N3) and rapid eye movement (REM) sleep, total sleep time (TST), and time in bed (TIB). Sleep spindles and SWA were analyzed at C3 referenced to A2. Slow (12–14 Hz) and fast (14–16 Hz) spindle density (spindles per minute) analysis was performed on all NREM epochs with an automated algorithm [11] on the Wonambi Python package v5.24 (https://wonambi-python.github.io). EEG power spectral density analyses focused on SWA (0.6–4 Hz) using Welch’s method (Hamming window; 0.2 Hz bin resolution). This was performed on nonoverlapping 5 s epochs to calculate total and mean SWA summed across all NREM epochs.

### Statistical analysis

Memory measures at each confidence level (certain, somewhat certain, and guess) were corrected for response bias (correct − incorrect) and assessed separately. A fourth measure that included all correct responses (overall memory) was also tested. These were analyzed via 2 × 4 mixed ANOVA with group as the between-subject factor (wake and sleep) and test as the within-subjects factor (T1, T2, T3, and T4). Independent samples *t*-tests compared groups at each test. These tests alone could not address our three experimental questions, since each test conflates performance of the preceding tests (e.g. a significant group difference after relearning at T3 could indicate an effect of sleep on relearning, or the continuation of an effect of sleep on consolidation observed at T2). To correct for this and directly test our hypotheses, memory at each test was contrasted to produce measures of consolidation (T2 − T1), relearning (T3 − T2), and long-term memory (T4 − T1).

A similar ANOVA was used to analyze PVT lapses. Independent samples *t*-tests compared groups for all planned comparisons, including memory, age, cognitive tests, sleep questionnaires, actigraphy, and pretest performance. Spearman’s rho correlations examined the relationship between memory and sleep characteristics. Effect sizes are reported with partial eta squared (*ηp*^2^) and Cohen’s D (*d*). All statistical tests were two-tailed, significance level *p* < 0.05.

## Results

### Cognitive tests

Independent *t*-tests determined that groups did not differ in forward digit span (*t*(50) = 0.72, *p* = 0.48), backward digit span (*t*(50) = 0.47, *p* = 0.64), verbal learning for RAVLT trial 7 (*t*(49) = 0.68, *p* = 0.50), RAVLT trial 8 (*t*(49) = 0.32, *p* = 0.75), and fluid intelligence (*t*(50) = 0.16, *p* = 0.87; Supplementary Table S1). Groups were therefore comparable on these cognitive tests.

### Factual knowledge task

A pretest performed prior to learning found little prior overall memory of the to-be-learned materials (Wake *M* = 52.4, *SD* = 7.3%; Sleep *M* = 53.0, *SD* = 7.4%) and no significant difference between groups (*t*(50) = 0.28, *p* = 0.78, *d* = 0.08).

**Figure 2.**
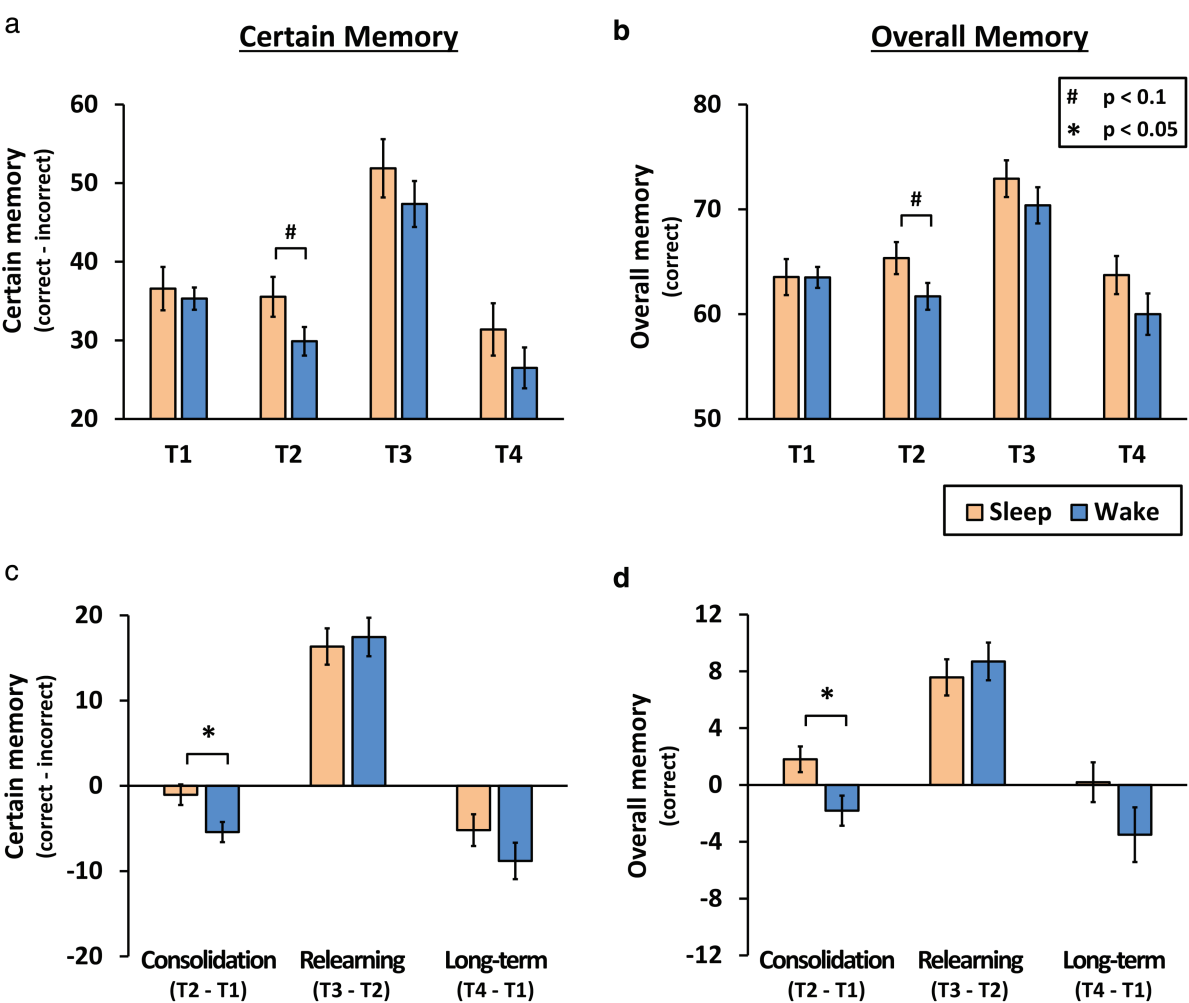
Results for certain and overall memory. (A) Performance at each test for memories rated as certain and (B) memory scores overall. (C) For certain memories, the sleep group remembered significantly more than the wake group after the retention interval (consolidation), indicating sleep-dependent consolidation of factual knowledge. However there was no significant difference between the two groups after relearning or after 1 week. (D) Overall memory scores showed the same pattern. Mean ± SEM.

Performance for certain and overall memory at each test are depicted in Table 3 and Figure 2. Consistent with prior studies using this task [24, 25], group differences were expected for certain memories (correct − incorrect) because this measure accounts for individual participants’ response bias and is the least prone to guessing. A mixed ANOVA with group (sleep and wake) and test (T1, T2, T3, and T4) showed a main effect of test (*F*(3, 150) = 40.14.2, *p* < 0.001, *ηp*^2^ = 0.45), but no effect of group (*F*(1, 50) = 1.61, *p* = 0.21, *ηp*^2^ = 0.03) and no interaction (*F*(3,150) = 1.53, *p* = 0.21, *ηp*^2^ = 0.03). Importantly, groups did not differ at T1 (*t*(50) = 0.41, *p* = 0.68, *d* = 0.11), which indicates there were no time-of-day effects when performing in the evening (sleep group) or the morning (wake group). There was a trend for better memory of the sleep group after the sleep period at T2 (*t*(50) = 1.81, *p* = 0.08, *d* = 0.5), but not after relearning at T3 (*t*(50) = 0.96, *p* = 0.34, *d* = 0.27) or after 1 week at T4 (*t*(50) = 1.16, *p* = 0.25, *d* = 0.32).

**Table 3.** Memory performance for each test

	Sleep		Wake			
	Mean	*SD*	Mean	*SD*	*t*	*P*
Overall						
T1	63.54	8.77	63.5	5.12	0.02	0.99
T2	65.35	7.78	61.69	6.52	1.84	0.07
T3	72.92	8.91	70.38	8.81	1.03	0.31
T4	63.73	9.27	60.00	10.08	1.39	0.17
Certain (correct − incorrect)						
T1	36.58	14.07	35.31	7.2	0.41	0.68
T2	35.54	12.92	29.88	9.27	1.81	0.08
T3	51.88	18.88	47.35	14.90	0.96	0.34
T4	31.38	16.97	26.5	13.27	1.16	0.25

*SD*, standard deviation

In order to directly test our hypotheses, we next created change scores between tests for consolidation (T2-T1), relearning (T3-T2), and long-term memory (T4-T1). Independent *t*-tests of these three measures revealed significantly greater memory consolidation for the sleep relative to wake group in the form of reduced forgetting (*t*(50) = 2.59, *p* = 0.01, *d* = 0.72; Figure 2, C), but unexpectedly there were no significant group differences for relearning (*t*(50) = 0.53, *p* = 0.72, *d* = 0.10) or long-term memory (*t*(50) = 0.74, *p* = 0.21, *d* = 0.35).

The overall memory measure is noisier because it includes responses that were somewhat certain or guesses, but it provides an overview of performance irrespective of confidence. Results were similar when considering overall memory (Figure 2, B), with a main effect of test (*F*(3, 150) = 40.14, *p* < 0.001, *ηp*^2^ = 0.45), but no effect of group (*F*(1, 50) = 1.61, *p* = 0.21, *ηp*^2^ = 0.03) and no interaction (*F*(3,150) = 1.53, *p* = 0.21, *ηp*^2^ = 0.03). Independent *t*-tests again revealed a trend for a sleep group advantage at T2 (*t*(50) = 1.84, *p* = 0.07, *d* = 0.51) but no other test (T1: *t*(50) = 0.02, *p* = 0.99, *d* < 0.01; T3: *t*(50) = 1.03, *p* = 0.31, *d* = 0.29; T4: *t*(50) = 1.39, *p* = 0.17, *d* = 0.39).

Our measures for consolidation, relearning and long-term memory showed a significant consolidation advantage for the sleep group (*t*(50) = 2.60, *p* = 0.01, *d* = 0.72; Figure 2, D), but no group differences for relearning (*t*(50) = 0.61, *p* = 0.55, *d* = 0.17) or long-term memory (*t*(50) = 1.55, *p* = 0.13, *d* = 0.43).

**Figure 3.**
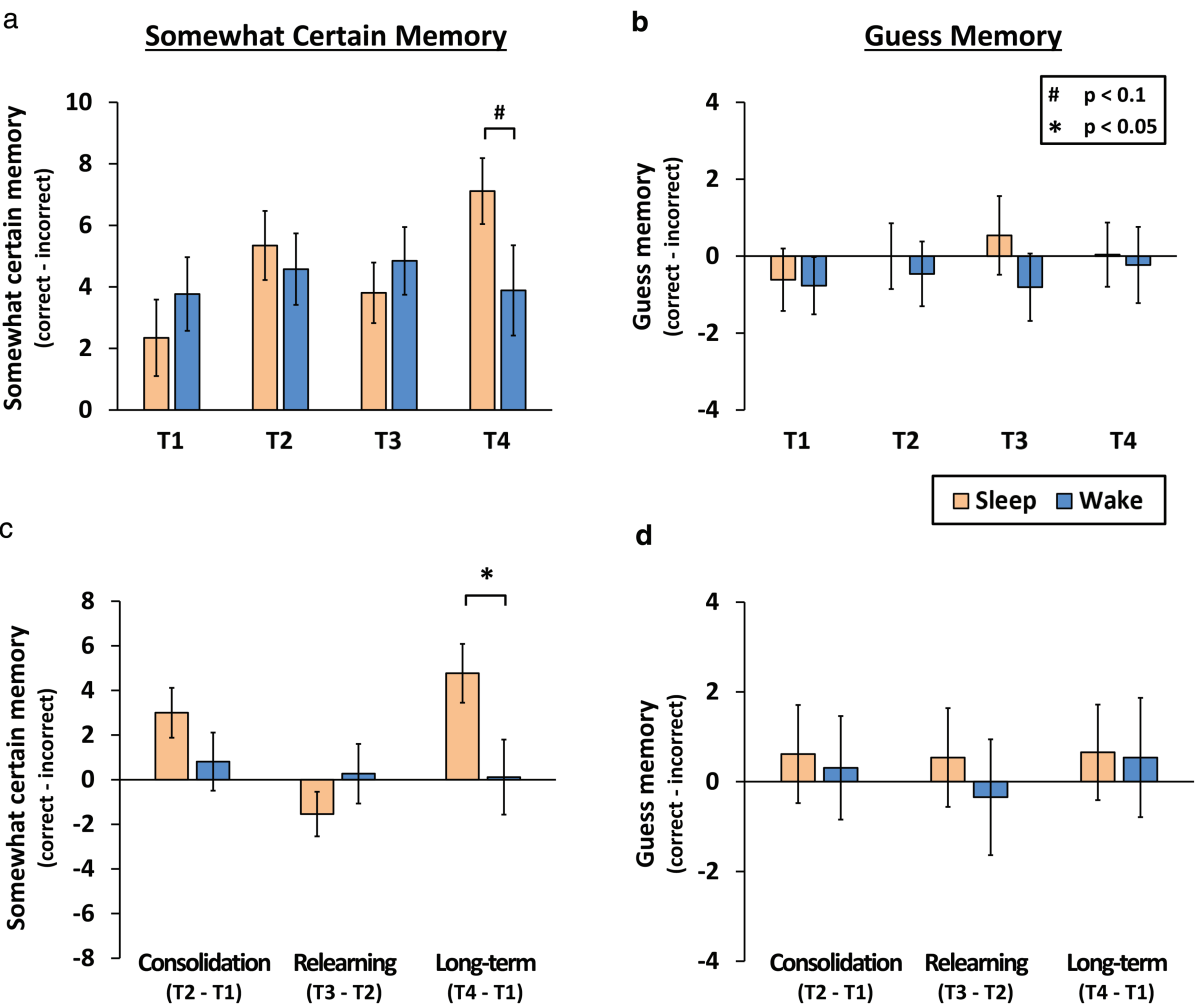
Results for somewhat certain memories and guesses. (A) Performance for memories rated as somewhat certain and (B) guesses. (C) For somewhat certain memories, there was no significant sleep advantage after the retention interval (consolidation), or after relearning, but there was a significant long-term memory benefit after 1 week. (D) Guess memory scores were close to chance and showed no group effects. Mean ± SEM.

For completeness, we also analyzed somewhat certain and guess responses. Prior studies using this paradigm showed poor performance and no effects related to sleep for these two measures [24–26], therefore we only predicted reliable group differences for the certain memory measure. Consistent with this, corrected responses (correct − incorrect) for guess and somewhat certain memory were very low (guess range = −0.81 to 0.54; somewhat certain range = 2.35 to 7.12), indicating inaccurate memory at these levels of certainty (Figure 3; Supplementary Table S2).

A mixed ANOVA including group and test for somewhat certain memory showed no group effect (*F*(1, 50) = 0.12, *p* = 0.75, *ηp*^2^ < 0.01), but there were trends for a main effect of test (*F*(3, 150) = 2.52, *p* = 0.06, *ηp*^2^ = 0.05), and for the interaction (*F*(3,150) = 2.57, *p* = 0.06, *ηp*^2^ = 0.05). Groups did not differ significantly at any individual test (*p* > 0.05), but there was a trend for a sleep advantage at T4 (*t*(50) = 1.78, *p* = 0.08, *d* = 0.49).

Moreover, groups did not significantly differ for measures of consolidation (*t*(50) = −1.27, *p* = 0.21, *d* = 0.35) or relearning (*t*(50) = 1.08, *p* = 0.28, *d* = 0.30), but the sleep group possessed significantly better memory for the long-term measure (*t*(50) = 2.18, *p* = 0.03, *d* = 0.60). While we made no specific predictions with regard to this measure, this finding is consistent with a benefit of sleeping between learning sessions for long-term memory.

Guesses showed no significant main effects or interactions (group, *F*(1, 50) = 0.63, *p* = 0.43, *ηp*^2^ = 0.01; test, *F*(3, 150) = 0.22, *p* = 0.88, *ηp*^2^ < 0.01; group*test, *F*(2,150) = 0.21, *p* = 0.89, *ηp*^2^ < 0.01), no group differences at any test or for measures of consolidation, relearning, or long-term memory (*p* > 0.05).

To summarize, we found evidence that overnight sleep benefited the consolidation of factual knowledge (certain memory) with a large effect size (*d =* 0.72), but not relearning of the same materials after sleep (*d* = 0.1). After 1 week, there was a small nonsignificant benefit of sleeping between the two learning sessions for our primary “certain memory” measure (*d* = 0.35), and a significant advantage for our secondary measure of “somewhat certain” memory (*d* = 0.60). Taken together, this may indicate a small to medium long-term advantage associated with sleep that our sample was too small to detect.

### Psychomotor vigilance

To capture levels of vigilance throughout the experiment, participants performed a PVT prior to commencement of learning in the first session (L1) and prior to each test thereafter (T1, T2, T3, and T4). A mixed ANOVA with group and time (L1, T1, T2, T3, and T4) found no significant effect of group on lapses (*F*(1, 39) = 0.13, *p* = 0.73, *ηp*^2^ < 0.01), no effect of time (*F*(4, 156) = 1.40, *p* = 0.24, *ηp*^2^ = 0.04), and no interaction (*F*(4, 156) = 0.47, *p* = 0.76, *ηp*^2^ = 0.01; *n* = 11 corrupted data points). Planned comparisons revealed no significant differences between groups at any point during the study (*p* > 0.05), indicating that levels of sustained attention during tests were unlikely to account for the group differences we observed.

### Actigraphy

Groups did not differ in actigraphically assessed TIB or TST for the mean of 3-days prior to commencement of the study (TIB: Wake *M* = 7.81, *SD* = 0.49 h; Sleep *M* = 8.01, *SD* = 0.56 h; TST: Wake *M* = 6.63, *SD* = 0.85 h; Sleep *M* = 6.42, *SD* = 0.81 h) or during the 1 week retention interval (*p* > 0.05; Table 2). Thus, groups were similarly well rested prior to learning and did not differ in their opportunity for sleep-dependent consolidation prior to the final test (T4).

### Polysomnography

Table 4 details the nocturnal sleep characteristics of the sleep group. SWA and sleep spindles are proposed to be a marker of memory consolidation [5, 10–14], therefore we correlated consolidation for certain memory (T2-T1) with four sleep features: (1) NREM mean SWA (0.6–4 Hz), (2) NREM total SWA, (3) NREM slow spindle density (12–14 Hz), and (4) NREM fast spindle density (14–16 Hz). We also correlated the same memory measure with time spent in each stage of sleep. There were no significant associations between any of these measures (*p* > 0.05; Supplementary Table S3).

**Table 4.** Overnight sleep parameters for the sleep group

	Sleep time	
	Mean	*SD*
TIB (min)	473.44	15.60
TST (min)	425.56	32.91
Sleep onset latency (min)	21.79	27.60
N1 (min)	14.02	18.98
N2 (min)	205.46	35.20
N3 (min)	85.75	27.65
REM sleep (min)	120.33	39.96
N1 (%)	3.30	4.44
N2 (%)	48.58	9.22
N3 (%)	20.07	5.99
REM sleep (%)	28.06	8.43

*SD*, standard deviation; N1, stage 1 sleep; N2, stage 2 sleep; N3, slow-wave sleep; %, percent of TST.

## Discussion

This study addressed the benefits of sleeping between two learning sessions, in terms of memory consolidation, relearning, and long-term memory. While we found evidence that supported the well-established benefit of sleep for memory consolidation, we observed no improvement to subsequent relearning of the same materials after sleep. One week later, the memory advantage for the most confident (certain) memories was reduced and no longer significant for those who slept between learning sessions, but memories with lower confidence (somewhat certain) were significantly enhanced. This extends the sleep-dependent consolidation of declarative memories to a more ecologically valid paradigm, but indicates no effect of sleep on relearning, and suggests that further research is needed to assess the practical long-term benefit to organizing study sessions to occur around nocturnal sleep.

### Sleep and relearning

The interaction between sleep and relearning is relatively underexplored. Our findings agree with an earlier study, where the consolidation of Swahili-English word pairs was enhanced across a nocturnal sleep period, but sleep provided no memory advantage after a relearning phase or after 10 days [1]. By contrast, others found that sleep enhanced both the overnight consolidation (*p* = 0.003, *d* = 0.99), and the speed of relearning Swahili-French word pairs (*p* < 0.001, *d* = 2.00), and this memory benefit remained with very large effect sizes 1 week (*p* < 0.001, *d* = 2.93) and 6 months later (*p* < 0.001, *d* = 2.46) [4]. It is difficult to account for why such similar studies produced different results. Mazza and colleagues [4] speculated that the relearning effect may have been attenuated for Bell et al. [1] because performance was too high at the beginning of relearning, leaving little room for improvement. However, the same explanation is unlikely to account for our findings because performance was well below ceiling prior to relearning, and on par with our prior study using the same factual knowledge task where significant improvements could still be achieved [25]. Related to this, the strength of memories that are prioritized for sleep-dependent consolidation is currently debated [6, 29]. The relearning effect observed by Mazza and colleagues came after learning to a 100% criterion before the retention interval [4], therefore it is possible that memories formed in the current study were too weak to undergo the sleep-dependent reorganization that benefits subsequent relearning. One caveat to consider is that the statistical power of our study could only detect large effects. Our findings do not preclude a modest effect of sleep on relearning. However, this is unlikely given the very small observed effect size for relearning (*d* = 0.17) that was in the *opposite* direction to our prediction (i.e. the wake group marginally outperformed the sleep group; Figure 2, C). We propose that the effect of sleep on relearning that was observed in relation to vocabulary learning [4] is less relevant to learning of detailed factual knowledge.

The absence of a sleep-relearning effect was unexpected given prior indications that sleep may assist the formation of schema [12, 15, 16], and these schema might in turn facilitate subsequent learning [19, 20]. Our task—consisting of detailed facts about related species that were likely to form schema [12]—was well placed to examine these possibilities. A recent category learning study found that sleep enhanced relearning of multimodal category structure, but without the typical benefit to consolidation [18], indicating a more subtle reorganization of knowledge during sleep to facilitate relearning. Potentially, this restructuring of knowledge also occurred for arthropod species in our study, but it had no impact on participants’ capacity to improve their knowledge during relearning.

Whether or not these subtle design details can account for conditions when sleep impacts relearning or not, they highlight that caution must be taken when generalizing the benefits of sleep in laboratory-based tasks to declarative learning in general. Establishing the boundary conditions of these effects will be crucial for translation of this research to determine the optimal sleep-study schedules to produce enduring long-term memories.

### Sleep and consolidation

While sleep did not influence relearning, we did find that the well-established effect on consolidation translated to our more ecologically valid task. We were motivated to use a naturalistic paradigm by several recent studies that question the precise conditions under which sleep-dependent consolidation occurs [30]. For example, the stabilizing effect of sleep to reduce interference could not be replicated [31, 32], while instances of sleep reorganizing information to extract gist [16, 22, 33] and provide insight [34] have produced inconsistent results. Most consolidation studies use simple verbal memory tasks (e.g. word paired associates) that yield consistent sleep-dependent improvements [1–4], yet even these effects disappear when the information load during encoding becomes too high [35]. This suggestion that sleep-memory effects are highly task-dependent motivated us to explore the extent to which they generalize to classroom learning, by using a similar form of semantic learning material, information load and learning time period (1 h 40 min) to a typical class. Our results suggest that the declarative memory benefits of sleep are robust and generalizable. It is likely that our naturalistic task and more “process pure” memory tasks like paired-associate learning rely on similar processes of sleep-dependent consolidation and both are highly relevant to classroom learning.

Several theoretical frameworks broadly agree that these effects reflect an active process during sleep that reactivates, strengthens, and reorganizes memories into long-term storage [5, 15, 16]. Slow waves and spindles that occur during NREM sleep have been highlighted as potential mechanisms of this process [5, 10–14], although we found no evidence that these sleep features were associated with memory consolidation. These theories also agree that consolidation promotes a gradual loss of episodic context as memories are integrated into existing knowledge networks, but sleep’s role in this semanticization process remains poorly understood. Sleep may support several aspects of semantic memory, such as the abstraction of statistical rules [36] and the integration of new words into the mental lexicon [13]. However, other related functions like cross-modal concept learning may be more dependent on time rather than sleep [37]. Our unconstrained, naturalistic task lacks specificity to determine whether these features of semantic memory were influenced by sleep. While there is an obvious path from our hierarchical, fact-based stimuli to long-term semantic knowledge, further studies are required that more tightly control and measure aspects of semantic memory (e.g. the loss of episodic context) to determine the exact memory functions that underscore the sleep benefit we observed.

It is also likely that passive protection of memories from interference contributed to the sleep group advantage. Prior research on vocabulary learning observed sleep enhancements that were independent of the amount of interference during a retention interval [3], but potentially our complex stimuli are more vulnerable to interference. That is, sentences and images from the materials would overlap to a greater extent with information learned on other topics by the wake group during the retention interval, leading to interference and forgetting. Such interference will also be higher in our sample of students in higher education, and could be explored in future studies by manipulating the activities of the wake group.

### Sleep and long-term memory

Sleeping between learning sessions enhanced high confidence certain memories 1 week later with a medium effect size (*d* = 0.35) although this did not reach significance in our sample. There was, however, a significant benefit of sleep for lower confidence (somewhat certain) memories with a larger effect size (*d* = 0.60). No predictions were made for this measure because it typically has poor accuracy and no association with sleep [24–26] (Figure 3; Supplementary Table S2). Indeed, performance was poor for this measure and it did not show the initial consolidation effect associated with sleep that was predicted and observed for certain memories (T2-T1). Memories that are not rehearsed will grow weaker over time, therefore “certain memories” that were enhanced by sleep may have simply been weakened across the 1 week retention interval. In this way, the sleep advantage remained, but the general reduction in memory strength shifted this effect to the lower certainty measure.

Additionally, the numerical advantage for certain memories with a medium effect size may suggest the existence of a small to medium long-term effect for this measure that we lacked statistical power to detect. This conjecture is supported by a study using the same factual knowledge task, where sleeping between two learning episodes provided a significant “certain memory” improvement after one week, also with a medium effect size (*d* = 0.56) [25]. The key difference to the current study was that learning episodes occurred either side of a nap rather than a full night of sleep, but both forms of sleep enhance long-term memory [10, 14, 26, 38].

Some earlier findings indicated that obtaining nocturnal sleep immediately after learning was beneficial to memory measured after 36 h [3], but our findings suggest that after 1 week the sleep benefit diminished below the point where we could detect a large effect. The absence of a significant nocturnal sleep benefit after relatively long delays (i.e. 1 week or longer) is a consistent observation in the literature, where a handful of studies have found statistically significant long-term enhancements to memory [4, 39], but the majority have not (for review see [22]). These null findings, like our own, may be due to smaller effect sizes at longer delays. We observed increased variance at this test, which was in turn likely the result of individual differences in rates of forgetting that would contribute to a reduced effect size. It is also possible that there was recovery of consolidation during subsequent sleep opportunities [40], although the effects of recovery sleep on long-term memory have yet to be established empirically. In the current study, the wake group slept at home immediately after the second learning session, providing an opportunity to consolidate the materials and “recover” to some extent. Further studies with increased statistical power are necessary to firmly establish the extent to which sleep improves long-term memory, which would help determine whether organizing sleep and study sessions close to one another is practically relevant for learning.

### Sleep and encoding

Finally, the time-of-day that initial learning occurred in our study differed between groups, raising the possibility that alertness levels may have been different in the two groups and impacted upon their capacity to learn. This was unlikely to be a confounding factor, because psychomotor vigilance did not differ significantly between groups at any point during the experiment. More importantly, performance on the first test (T1) did not differ between groups, indicating that encoding of materials was similar whether learning took place in the morning or evening. Encoding has been found to be impaired after one night of total sleep loss [41] or several nights of partial sleep loss [24, 42], but the 14 h of wakefulness that our wake group experienced prior to learning had no negative effect on their capacity to learn. The similar performance levels at T1 also suggests that groups were well matched in terms of their learning ability, in agreement with the cognitive tests we administered prior to the main experiment.

## Conclusion

In sum, this study attempted to replicate some recent findings of sleep’s role in memory processing with a view to assess how these effects translate to learning in daily life. Our findings suggest that sleep can reduce forgetting of factual knowledge in the short term, but sleep has little impact on an individual’s capacity to relearn the same materials. This suggests that the effect of sleep on relearning may have been overestimated, and highlights that further translational research is needed to assess the relevance of sleep-memory findings to learning in education.

## Supplementary Material

zsaa210_suppl_Supplementary_Material
