# Supplementary material for: Sleep after learning aids the consolidation of factual knowledge, but not relearning
Source: Sleep. 2020 Oct 9;44(3):zsaa210. doi: 10.1093/sleep/zsaa210 (PMC7953205; doi:10.1093/sleep/zsaa210)
Supplement: zsaa210_suppl_Supplementary_Material [file zsaa210_suppl_supplementary_material.docx]

**Supplementary Materials**

**Sleep after learning aids the consolidation of factual knowledge,
but not relearning**

James N. Cousins^a,b^, Teck Boon Teo^a,c^, Zhi Yi Tan^a^, Kian F. Wong^a,c^,
& Michael W. L. Chee^a,c^

^a^Centre for Cognitive Neuroscience, Duke-NUS Medical School, Singapore, 169857

^b^Donders Institute for Brain, Cognition & Behaviour, Radboud University Medical Centre, 6525 EN, Nijmegen, The Netherlands

^c^Centre for Sleep and Cognition, Yong Loo Lin School of Medicine, National University of Singapore, Singapore

Materials & correspondence: Michael W.L. Chee

[michael.chee@nus.edu.sg](mailto:michael.chee@nus.edu.sg)

**Table S1. Cognitive test performance.**

**Table S2. Certainty responses for each test.**

**Table S3. The relationship between memory consolidation and sleep parameters.**

**Table S1. Cognitive test performance.**

|  | Sleep | | Wake | | *t* | *p* |
| --- | --- | --- | --- | --- | --- | --- |
|  | Mean | SD | Mean | SD |  |  |
| Raven’s Advanced Progressive Matrices score | 9.12 | 1.76 | 9.19 | 1.47 | 0.16 | 0.87 |
| Forward Digit span | 11.38 | 2.28 | 11.84 | 2.36 | 0.72 | 0.48 |
| Backward Digit Span | 8.42 | 2.21 | 8.69 | 1.95 | 0.47 | 0.64 |
| RAVLT Trial 6 | 12.73 | 1.97 | 12.32 | 2.34 | 0.68 | 0.50 |
| RAVLT Trial 7 (delayed recall) | 12.31 | 2.41 | 12.08 | 2.73 | 0.32 | 0.75 |

Note. SD = standard deviation; RAVLT = Rey Auditory Verbal Learning Task

**Table S2. Certainty responses for each test.**

|  | Sleep | | Wake | |
| --- | --- | --- | --- | --- |
|  | Mean | SD | Mean | SD |
| *Certain* |  |  |  |  |
| T1 Correct | 41.04 | 12.52 | 40.38 | 7.40 |
| Incorrect | 4.46 | 4.90 | 5.08 | 3.20 |
| T2 Correct | 40.50 | 12.39 | 34.88 | 8.47 |
| Incorrect | 4.96 | 5.50 | 5.00 | 4.27 |
| T3 Correct | 56.77 | 16.24 | 53.85 | 13.21 |
| Incorrect | 4.88 | 4.77 | 6.50 | 3.96 |
| T4 Correct | 36.04 | 15.87 | 33.35 | 10.73 |
| Incorrect | 4.65 | 5.48 | 6.85 | 5.15 |
| *Somewhat Certain* |  |  |  |  |
| T1 Correct | 12.00 | 4.29 | 12.38 | 5.46 |
| Incorrect | 9.65 | 4.80 | 8.62 | 4.06 |
| T2 Correct | 13.19 | 4.74 | 13.46 | 5.09 |
| Incorrect | 7.85 | 4.39 | 8.88 | 4.91 |
| T3 Correct | 9.92 | 5.79 | 10.65 | 6.00 |
| Incorrect | 6.12 | 4.54 | 5.81 | 3.86 |
| T4 Correct | 16.00 | 6.84 | 13.85 | 5.96 |
| Incorrect | 8.88 | 4.48 | 9.96 | 4.80 |
| *Guess* |  |  |  |  |
| T1 Correct | 9.88 | 5.16 | 10.08 | 5.59 |
| Incorrect | 10.50 | 6.43 | 10.85 | 4.41 |
| T2 Correct | 11.12 | 6.55 | 13.15 | 6.24 |
| Incorrect | 11.12 | 6.35 | 13.62 | 6.47 |
| T3 Correct | 5.46 | 7.13 | 5.50 | 4.69 |
| Incorrect | 4.92 | 4.06 | 6.31 | 5.64 |
| T4 Correct | 10.19 | 7.56 | 11.50 | 5.80 |
| Incorrect | 10.15 | 6.77 | 11.73 | 5.74 |
|  |  |  |  |  |

Note. SD = standard deviation

**Table S3. The relationship between memory consolidation and sleep parameters.**

|  | Spearmans Rho | |
| --- | --- | --- |
|  | r^s^ | p |
| *Certain memory consolidation (T2-T1)* |  |  |
| N1 (min) | -.22 | .30 |
| N2 (min) | .08 | .70 |
| N3 (min) | -.14 | .52 |
| Rapid-eye movement sleep (min) | -.004 | .99 |
| NREM Mean SWA | .21 | .33 |
| NREM Total SWA | .17 | .44 |
| NREM slow spindle density (12-14 Hz) | .18 | .43 |
| NREM fast spindle density (14-16 Hz) | .31 | .17 |

Note: T1 = Test 1; T2 = Test 2; N1 = stage 1 sleep; N2 = stage 2 sleep;
N3 = slow-wave sleep; NREM = non-rapid-eye movement sleep;

SWA = slow-wave-activity (0.5-4Hz); Spindle density = spindles per min
